# Clonal Parental Effects on Offspring Growth of Different Vegetative Generations in the Aquatic Plant *Pistia stratiotes*

**DOI:** 10.3389/fpls.2022.890309

**Published:** 2022-06-27

**Authors:** Li-Min Zhang, Sergio R. Roiloa, Jia-Fan Zhang, Wen-Han Yu, Chen-Yan Qiu, Dan-Hao Wang, Fei-Hai Yu

**Affiliations:** ^1^Institute of Wetland Ecology & Clone Ecology, Taizhou University, Taizhou, China; ^2^Zhejiang Provincial Key Laboratory of Plant Evolutionary Ecology and Conservation, Taizhou University, Taizhou, China; ^3^BioCost Group, Department of Biology, Faculty of Science, Universidade da Coruña, A Coruña, Spain

**Keywords:** clonal plant, nutrients, provisioning effect, ramet size, trans-generational effects

## Abstract

Parental (environmental) effects can modify the growth of offspring, which may play an essential role in their adaptation to environmental variation. While numerous studies have tested parental effects on offspring growth, most have considered offspring growth of only one generation and very few have considered offspring growth of different generations. We conducted a greenhouse experiment with an aquatic clonal plant *Pistia stratiotes*. We grew a single ramet of *P. stratiotes* under low or high nutrients, the initial (parent) ramets produced three different generations of offspring ramets, and these offspring ramets were also subjected to the same two nutrient levels. High nutrients currently experienced by the offspring increased biomass accumulation and ramet number of all three offspring generations of *P. stratiotes*. However, these positive effects on biomass were greater when the offspring ramets originated from the parent ramets grown under low nutrients than when they were produced by the parent ramets grown under high nutrients. These results suggest that parental effects can impact the performance of different offspring generations of clonal plants. However, heavier offspring ramets produced under high nutrients in parental conditions did not increase the subsequent growth of the offspring generations. This finding indicates that parental provisioning in favorable conditions may not always increase offspring growth, partly depending on root allocation but not ramet size such as ramet biomass.

## Introduction

The phenotype of a plant individual can be influenced by the environmental condition experienced by its parent, a phenomenon called parental effects or trans-generational effects (Agrawal, 2002; Mousseau et al., 2009; González et al., 2016; Waterman and Sultan, 2021). Parental effects can influence offspring not only *via* seeds produced through sexual reproduction (Bossdorf et al., 2010; Mondoni et al., 2014; Baker et al., 2019; Veselá et al., 2021) but also *via* ramets (asexual individuals) produced through clonal growth or vegetative reproduction (Latzel and Klimešov, 2010; Dong et al., 2018a; González et al., 2018; Portela et al., 2020). Both sexual and clonal parental effects have important ecological and evolutionary implications as they can potentially influence plant fitness, intraspecific and interspecific interactions, population dynamics, and community structure (Badyaev and Uller, 2009; Droste et al., 2010; Geng et al., 2016; Herman and Sultan, 2016; Pérez-Ramos et al., 2019).

Increasing evidence shows that parental effects may be adaptive, enhancing the fitness of the offspring when established in an environment similar to their parents (Herman et al., 2012; Rasmann et al., 2012; Latzel et al., 2014; Dong et al., 2017; Baker et al., 2018). For instance, when offspring of *Polygonum persicaria* grow in shade, offspring produced by shaded parents perform better than offspring produced by parents under sunlight (Baker et al., 2018). Adaptive parental effects have been reported in several plant species in response to a variety of biotic and abiotic factors (Lacey and Herr, 2000; Whittle et al., 2009; Dong et al., 2017; Waterman and Sultan, 2021). As a result, parental effects are recognized as an important source of phenotypic variation that may have an essential role in local adaptations (Herman and Sultan, 2011; Holeski et al., 2012; Dong et al., 2018a).

Many studies have shown that parental effects caused by differences in the quality of resources provisioned to offspring may be more likely to be adaptive because they can persist through the life cycle (Roach and Wulff, 1987; Herman and Sultan, 2011; Germain et al., 2013; Zas et al., 2013). For example, larger seedlings of *Pinus pinaster* can come from larger seeds that are produced in favorable parental environments (Zas et al., 2013). Compared to sexual propagules (e.g., seeds), vegetative propagules (e.g., ramets) of clonal plants are larger in size and mass, and thus their potential for resource provisioning may be relatively high (Dong et al., 2019). For instance, González et al. (2017) found that parent ramets of *Trifolium repens* grown in better conditions produced larger/heavier vegetative propagules that enabled offspring to grow better. Dong et al. (2018a, 2019) and Portela et al. (2020) showed that clonal fragments of *Alternanthera philoxeroides* produced in favorable parental environments benefited the subsequent growth of clonal offspring. These studies suggest that resource provisioning is one of the most important mechanisms underlying clonal parental effects (González et al., 2017; Dong et al., 2018a, 2019; Portela et al., 2020).

A high proportion of aquatic species are clonal and capable of rapid vegetative reproduction (Sosnová et al., 2010; Wang et al., 2016; Zhang et al., 2019; Adomako et al., 2021). A clone often consists of a large network of interconnected ramets belonging to different vegetative generations (Dong, 2011). However, previous studies testing clonal parental effects on offspring performance have involved clonal offspring of only a single vegetative generation (Dong et al., 2017, 2019; González et al., 2017; Portela et al., 2020), and have not considered the potential differences among offspring ramets of different vegetative generations. As the size and biomass of offspring ramets commonly become smaller with increasing vegetative generation (Wang et al., 2014), their ability of resource provisioning may become weaker. Therefore, the magnitude of clonal parental effects on offspring performance may differ when offspring of different vegetative generations are considered.

To test how clonal parental effects influence the offspring growth of different vegetative generations, with a focus on provisioning as a possible mechanism, we conducted a greenhouse experiment on an aquatic clonal plant *Pistia stratiotes*. We grew a single ramet of *P. stratiotes* under low or high nutrients, and its offspring ramets of three different generations, i.e., primary, secondary, and tertiary offspring ramets were also subjected to low or high nutrients. Specifically, we tested three hypotheses: (1) clonal parental effects would impact offspring growth of all three vegetative generations of *P. stratiotes*; (2) for all three vegetative generations, offspring produced by the parent ramet under high nutrients would perform better than offspring produced by the parent ramet under low nutrients, because providing high nutrients to the parent ramet may allow them to produce high-quality clonal offspring; and (3) the magnitude of clonal parental effects on offspring performance would become smaller with increasing the vegetative generation, i.e., parental effects would be the highest on the growth of the primary ramets, the lowest on that of the tertiary ramets, and in between on that of the secondary ramets.

## Materials and Methods

### Study Species and Material Preparation

*Pistia stratiotes* L. (water lettuce, Araceae) is an aquatic, free-floating, stoloniferous clonal plant (Pettet and Pettet, 1970). This species is native to South America and is now widely spread in tropical and subtropical regions of the world (Yang et al., 2014; Galal et al., 2019). Rosette leaves come out from nodes of the highly compressed stems and adventitious roots arise at the base of the rosette. *Pistia stratiotes* are capable of rapid clonal growth (Adomako et al., 2021) and stolons grow out from the leaf axils to form offspring ramets (Odjegba and Fasidi, 2004). The species is listed as an invasive species in the Global Invasive Species Database (http://issg.org/database/welcome/) because they can form extensive floating mats that block the air-water interface, reduce oxygen levels in the water, and decrease biodiversity (Adebayo et al., 2011; Galal et al., 2019). However, *P. stratiotes* have been shown to have the potential for the management of water quality due to their ability to accumulate heavy metals from water (Hanks et al., 2015; Adomako et al., 2020).

On June 12, 2020, ramets of *P. stratiotes* were collected from Yongning River (28°40′3″N, 121°23′4″E) in Taizhou, Zhejiang Province, China, and brought to a tank (95 cm in diameter × 60 cm in height) in a greenhouse at Taizhou University for propagation. After 3 weeks, all ramets had produced new offspring ramets. On July 3, 2020, 30 new offspring ramets of similar size were selected and their stolons, if any, were removed. Of the 30 ramets, ten were randomly selected, dried at 70°C for 48 h, and weighed to measure initial dry mass (mean ± SE: 1.95 ± 0.19 g). The remaining 20 ramets (hereafter referred to as parent ramets) were used for the experiment described below.

### Experimental Design

**Figure 1:**
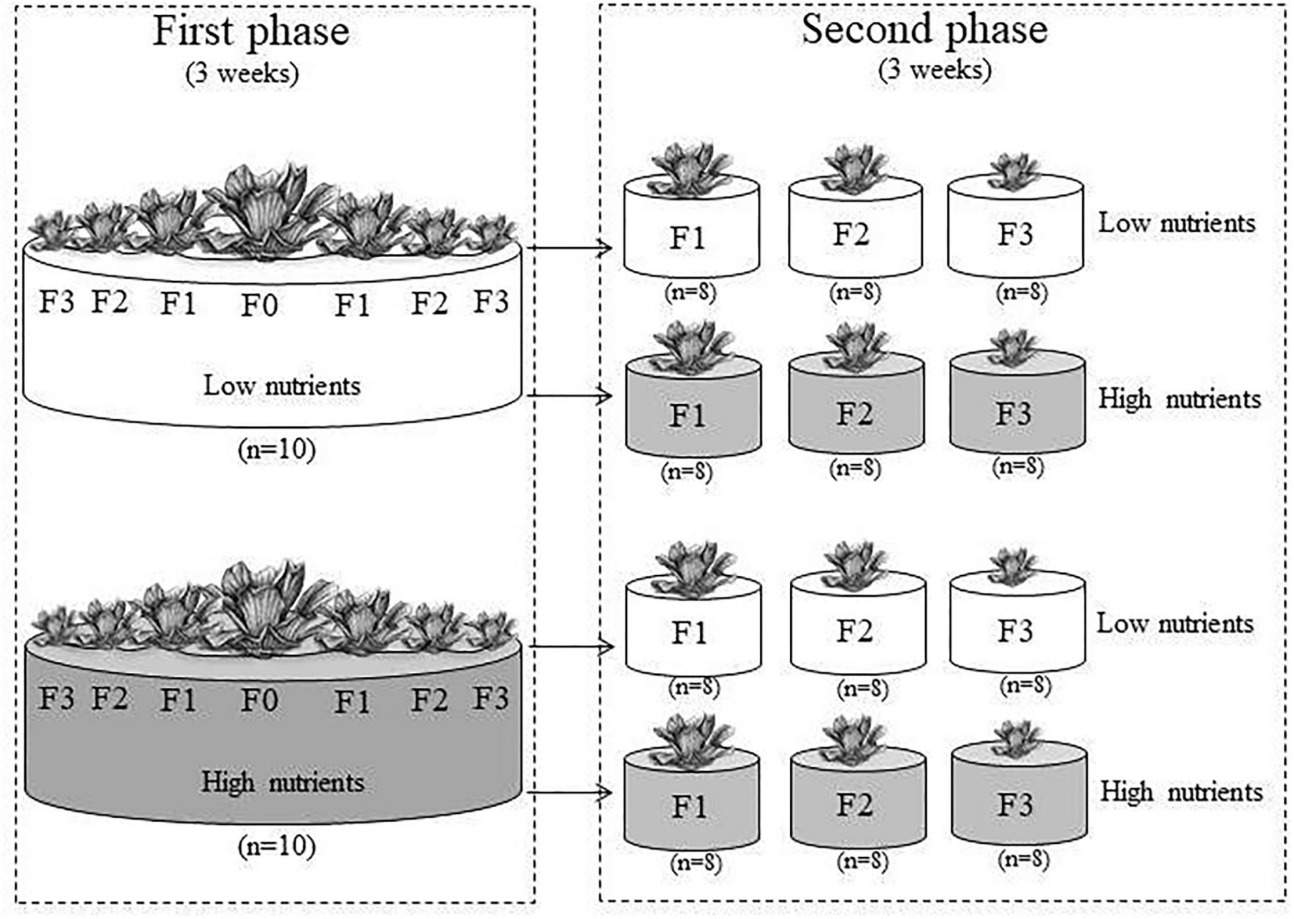
Scheme of the experimental design. In the first phase, parent ramets (F0) produced F1, F2, and F3 offspring ramets under low or high nutrient levels. In the second phase, the F1, F2, and F3 offspring ramets from the first phase were also subjected to the same two nutrient levels.

The experiment consisted of two phases. In the first phase, parent ramets (F0) were randomly subjected to two nutrient levels, and the primary (F1) offspring ramets (i.e., daughter ramets of the parent ramets), secondary (F2) offspring ramets (i.e., daughter ramets of the F1 ramets or granddaughter ramets of the parent ramets), and tertiary (F3) offspring ramets (i.e., daughter ramets of the F2 ramets or granddaughter ramets of the F1 ramets or grand-granddaughter ramets of the parent ramets) from each parent ramet were harvested (Figure 1). In the second phase, the F1, F2, and F3 ramets originating from the F0 ramets grown at each nutrient level in the first phase of the experiment were subjected to the same two nutrient levels (Figure 1).

The first phase of the experiment started on July 3, 2020. We randomly assigned the 20 parent ramets to two nutrient levels (high vs. low), and each treatment had ten replicates. For the high and low nutrient levels, the parent ramets were grown individually in containers (65 cm in diameter × 44 cm in height) filled with 70 L of 30% and 3% Hoagland solution, respectively. The Hoagland solution contained 945 mg/L of Ca(NO_3_)_2_·4H_2_O, 506 mg/L of KNO_3_, 80 mg/L of NH_4_NO_3_, 136 mg/L of KH_2_PO_4_, 493 mg/L of MgSO_4_, 13.9 mg/L of FeSO_4_·7H_2_O, and 18.7 mg/L of EDTA·2Na. Every week, 2 L of 30% or 3% Hoagland solution was added to each container to compensate for nutrient depletion by the plants and, if needed, additional water was added to each container to compensate for water loss due to evaporation. The experiment was conducted in an open area on the campus of Taizhou University. The first phase of the experiment ended on July 24, 2020 and lasted for 3 weeks. At harvest, each parent ramet had produced 12–29 F1 offspring ramets, 20–121 F2 offspring ramets, and 2–135 F3 offspring ramets.

The second phase of the experiment began on July 24, 2020. We selected two F1, two F2, and two F3 ramets from each container (produced by each parent ramet) at the end of the first phase of the experiment, and, thus, obtained 20 offspring ramets of each generation (F1, F2, and F3) from each of the two nutrient levels (high vs. low). Of the 20 offspring ramets of each generation from each nutrient level, 16 were randomly selected and assigned to the two nutrient treatments as described in the first phase, and the remaining four, which were not used in the second phase, were dried at 70°C for 48 h and weighed to measure initial biomass. Initial biomass of the F1, F2, and F3 ramet were 0.723 ± 0.133 g, 0.249 ± 0.055 g, 0.026 ± 0.004 g (mean ± SE), respectively, from the low nutrient level, and 1.328 ± 0.317 g, 0.454 ± 0.057 g, and 0.101 ± 0.016 g, respectively, from the high nutrient level. We selected heavier ramets under high nutrients than under low nutrients of *P. stratiotes* because a previous study found that high nutrients increased biomass per ramet of *P. stratiotes* (Adomako et al., 2021).

In the second phase, each treatment had eight replicates, resulting in a total of 96 containers (36 cm in diameter × 32 cm in height). Containers filled with 15 L of 30% and 3% Hoagland solution for the high and the low nutrient treatment, respectively. Every week, 1 L of 30% or 3% Hoagland solution was added to each container to compensate for nutrient depletion by the plants and, if needed, additional water was added to each container to compensate for water loss due to evaporation.

The second phase of the experiment ended after 3 weeks, on August 14, 2020, when plants in most treatments had occupied the whole water surface in the containers. Photosynthetic photon flux density at the water surface at noon was 632–1,806 μmol m^−2^ s^−1^, as measured weekly with a quantum sensor (LI-250 A; LI-COR Biosciences). The daily mean air temperature was 29.1°C, and the mean relative humidity was 80.3%, each measured hourly with Hygrochron temperature loggers (iButton DS1923; Maxim Integrated Products, USA).

### Harvest and Measurements

At the end of the first phase of the experiment, after the selection of the two F1, F2, and F3 ramets for the second phase of the experiment, the remaining parts of the plants in each container were harvested. We recorded the number of the F1, F2, and F3 ramets separately for each container. Then, the parent ramet, as well as the F1, F2, and F3 offspring ramets were separately dried at 70°C for 72 h and weighed to obtain biomass. Biomass per ramet (i.e., final total dry mass/number of ramets) was calculated for F1, F2, and F3 offspring ramets, respectively. At the end of the second phase of the experiment, we counted the number of all ramets in each container. Then, the plants in each container were dried at 70°C for 72 h and weighed to measure biomass.

### Data Analysis

For the first phase of the experiment, we used a *t*-test to examine the differences in biomass and number of the F1, F2, and F3 ramets, as well as biomass per F1, F2, and F3 ramet of *P. stratiotes*. For the second phase of the experiment, we employed two-way ANOVAs to test the effects of parental nutrient level, offspring nutrient level, and their interaction on biomass and the number of ramets of *P. stratiotes* produced by the offspring ramets of each generation, i.e., the F1, F2, and F3 offspring ramets produced by the parent ramets in the first phase of the experiment. Before analysis, biomass and number of ramets derived from the F2 offspring ramets and number of ramets derived from the F3 offspring ramets were ln-transformed to remove heteroscedasticity and to increase normality; figures show untransformed data. Statistical analyses were carried out with SPSS 22.0 (IBM Corp., Armonk, New York, USA). Plants in three containers (one F1 ramet produced by the parent ramet under low nutrients in the first phase of the experiment and grown under low nutrients in the second phase, one F2 ramet produced by the parent ramet under high nutrients in the first phase and grown under high nutrients in the second phase, and one F3 ramet produced by the parent ramet under high nutrients in the first phase and grown under high nutrients in the second phase) were damaged by herbivores during the experiment and were thus excluded from harvest and analysis.

## Results

### Effects of Nutrients on Offspring Performance in the First Phase

**Figure 2:**
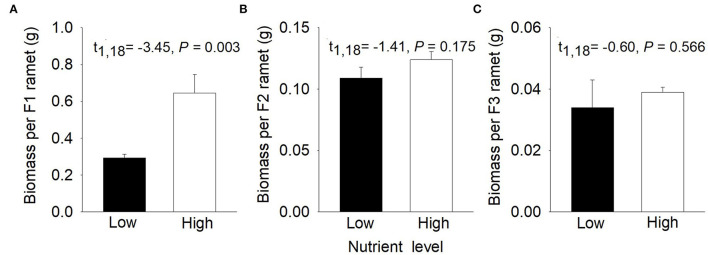
Effects of nutrient level (low vs. high) on **(A)** biomass per F1 ramet, **(B)** biomass per F2 ramet, and **(C)** biomass per F3 ramet of *Pistia stratiotes* in the first phase. Bars show means + SE (*n* = 10).

Compared to low nutrients, high nutrients significantly increased biomass per F1 ramet of *P. stratiotes* but had little effect on biomass per F2 ramet and biomass per F3 ramet (Figure 2). Total biomass and number of the F1, F2, and F3 offspring ramets were significantly greater under high than under low nutrients (Supplementary Figure S1).

### Effects of Parental and Offspring Nutrients on Offspring Performance in the Second Phase

**Figure 3:**
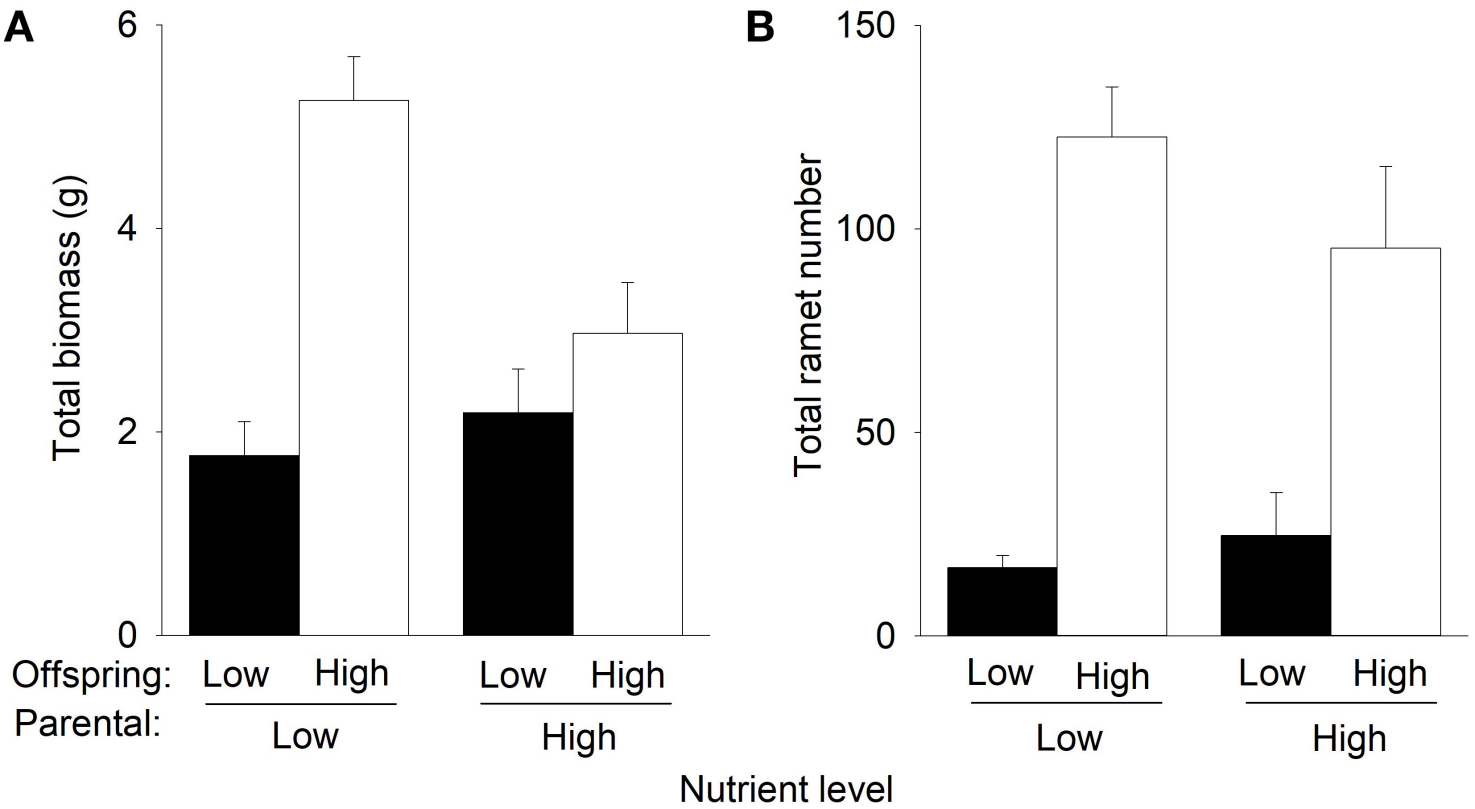
Effects of parental nutrient level and offspring nutrient level on **(A)** total biomass and **(B)** total ramet number of *Pistia stratiotes* produced by the F1 offspring ramets in the second phase of the experiment. Bars show means + SE.

Compared to low nutrients currently experienced by offspring, offspring high nutrients significantly increased total biomass and the number of F1 offspring ramets of *P. stratiotes* in the second phase (Table 1, Figure 3). However, such an effect on total biomass was much greater when the F1 offspring ramets were produced by the parent ramets grown under low nutrients than when they were produced by the parent ramets grown under high nutrients in the first phase (Figure 3A), as indicated by the significant interactive effect of parental nutrient level × offspring nutrient level (Table 1). Also, compared to parental low nutrients, parental high nutrients tended to increase total biomass (by 23.7%) of *P. stratiotes* under offspring low nutrients but markedly decreased it (by 43.5%) under offspring high nutrients (Figure 3A, Table 1). The parental nutrient level had no significant effect on the number of F1 offspring ramets of *P. stratiotes* in the second phase (Figure 3B, Table 1).

**Table 1:** ANOVA results for effects of parental nutrient level, offspring nutrient level, and their interaction on total biomass and total number of ramets of *Pistia stratiotes* produced by the (A) F1, (B) F2, and (C) F3 offspring ramets.

	**Total biomass**		**Total number of ramets**	
	***F*** **_1, 27_**	***P***	***F*** **_1, 27_**	***P***
**(A) F1 offspring ramets**				
**Parental nutrient level (P)**	5.66	**0.025**	0.52	0.479
**Offspring nutrient level (O)**	29.45	**<0.001**	42.86	**< 0.001**
**P × O**	11.88	**0.002**	1.70	0.203
**(B) F2 offspring ramets**				
**Parental nutrient level (P)**	9.73	**0.004**	0.03	0.877
**Offspring nutrient level (O)**	25.48	**<0.001**	70.52	**<0.001**
**P × O**	4.75	**0.038**	9.13	**0.005**
**(C) F3 offspring ramets**				
**Parental nutrient level (P)**	1.55	0.224	1.48	0.234
**Offspring nutrient level (O)**	18.84	**<0.001**	34.11	**<0.001**
**P × O**	4.92	**0.035**	0.36	0.555

*The bold values indicates P < 0.05*.

**Figure 4:**
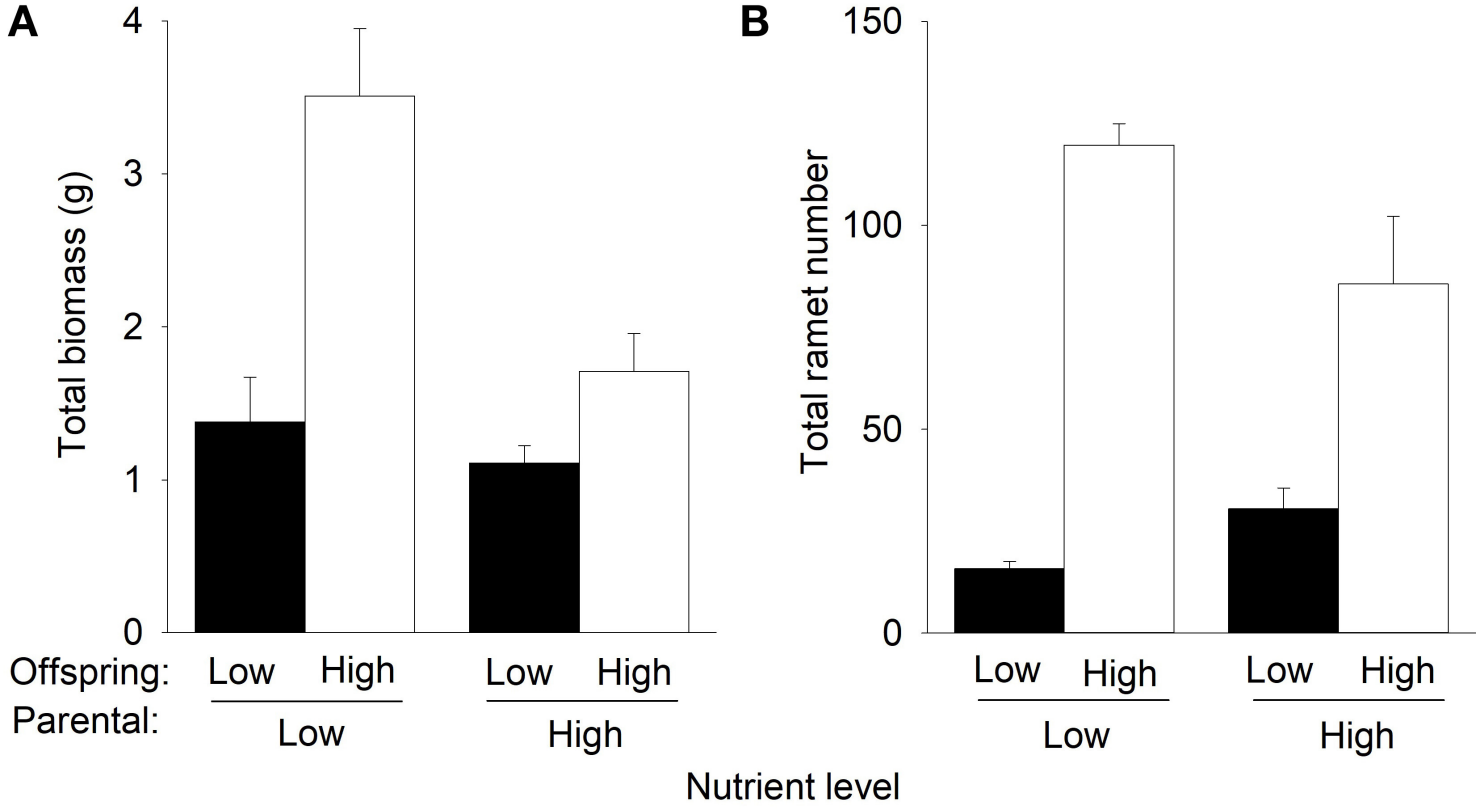
Effects of parental nutrient level and offspring nutrient level on **(A)** total biomass and **(B)** total ramet number of *Pistia stratiotes* produced by the F2 offspring ramets in the second phase of the experiment. Bars show means + SE.

Compared to offspring low nutrients, offspring high nutrients increased total biomass and the number of the F2 offspring ramets of *P. stratiotes* in the second phase (Table 1, Figure 4). However, such an effect on total mass and number of ramets was much greater when the F2 offspring ramets were produced by the parent ramet grown under low nutrients than when they were produced by the parent ramets grown under high nutrients in the first phase (Figure 4), as indicated by the significant interactive effect of parental nutrient level × offspring nutrient level (Table 1). Also, under offspring's low nutrients, the parental nutrient level had little effect on total biomass of *P. stratiotes*, but under offspring high nutrients, parental high nutrients markedly decreased it (by 51.4%) compared to parental low nutrients (Figure 4A, Table 1). Parental high nutrients increased the number of the F2 offspring ramets (by 92.1%) under offspring low nutrients but decreased it (by 25.9%) under offspring high nutrients (Figure 4B, Table 1).

**Figure 5:**
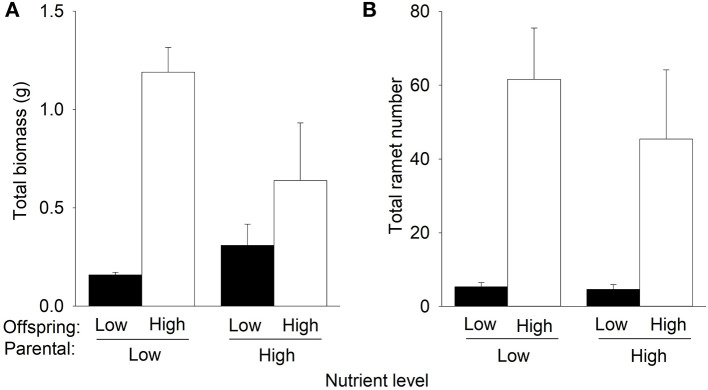
Effects of parental nutrient level and offspring nutrient level on **(A)** total biomass and **(B)** total ramet number of *Pistia stratiotes* produced by the F3 offspring ramets in the second phase of the experiment. Bars show means + SE.

Offspring high nutrients increased total biomass and the number of the F3 offspring ramets of *P. stratiotes* in the second phase (Table 1, Figure 5). However, such an effect on total biomass was much higher under parental low nutrients than under parental high nutrients (significant interactive effect of parental nutrient level × offspring nutrient level in Table 1, Figure 5A). The parental nutrient level did not significantly affect the number of the F3 offspring ramets in the second phase (Table 1, Figure 5B). Parental high nutrients increased total biomass (by 82.9%) under offspring low nutrients but decreased it (by 52.2%) under offspring high nutrients (Figure 5A, Table 1).

## Discussion

As expected, both in the first and the second phase, current high nutrients increased the production of biomass and the number of new offspring ramets of *P. stratiotes*, agreeing with many previous findings on other aquatic plants (Zhao et al., 2006; Jampeetong and Brix, 2009; Zhang et al., 2020). Besides the direct positive effect of high nutrients on the growth of offspring ramets, the benefit was also from physiological integration from their parent ramets growing under the favorable conditions of high nutrient availability. Physiological integration can allow acropetal transport of resources from well-established ramets to developing offspring. Benefits from physiological integration have been repetitively reported by various previous studies (e.g., Slade and Hutchings, 1987; Saitoh et al., 2002; Roiloa and Retuerto, 2006; Dong et al., 2015; Elgersma et al., 2015; Portela et al., 2021; Wang et al., 2021). In our study, it is logical to anticipate that the benefit gained by offspring connected to parents with high nutrient availability was due to the support received from their parents growing under favorable conditions.

High nutrients increased ramet number of different offspring generations but had different effects on ramet weight (i.e., biomass per ramet). Previous studies found that plants produced lighter and likely smaller ramets in response to high-density environments and produced heavier and, likely, also bigger ramets in response to low-density environments (Wang et al., 2014; Adomako et al., 2021). Therefore, the relationship between ramet biomass and ramet number was caused by the different responses to plant density (Wang et al., 2014). In our study, high nutrients increased the biomass of the F1 offspring ramets but had little effect on the biomass of the F2 and the F3 ramets. This is mainly because plants did not occupy the whole surface of containers when producing the F1 ramets, so high nutrients increased both ramet biomass and number under the low-density environment. However, the high nutrients increased the number of the F2 and the F3 ramets, resulting in increased plant density and intensity of intraspecific competition, which may limit the growth of the F2 and the F3 ramets. Thus, ramet biomass of the F2 and the F3 ramets was not significantly greater under high nutrients than in low nutrients.

The interaction of parental nutrient level and offspring nutrient level significantly affected the growth of different offspring generations. Compared to low nutrients experienced by the parent ramet, parental high nutrients inhibited subsequent growth of offspring in high nutrients for all three offspring generations but increased growth of the F3 offspring ramets in low nutrients. These results support the first hypothesis that parental effects could impact offspring growth of different vegetative generations of clonal plants. Similarly, previous studies have shown that parental environment effects can persist across vegetative generations in clonal plants (e.g., González et al., 2017; Dong et al., 2018b; Portela et al., 2020; Zhang et al., 2021). Our results suggest that paternal effects are fast in clonal plants as the vegetative reproduction is rapid.

The selected offspring ramets from the first phase that grew under high nutrients were relatively bigger and had nearly two times greater initial mass than did offspring taken from the first phase grew under low nutrients. However, such a sizeable advantage of ramets inhibited subsequent growth of offspring in high nutrients for all three vegetative generations. Contrary to the second prediction, the benefit obtained by offspring generations (F1, F2, and F3) growing in high nutrients was significantly higher when their parent ramets grew in low nutrients. A plausible explanation for this unexpected result could be based on biomass partitioning responses. As predicted by the optimal partitioning theory (Bloom et al., 1985; Hilbert, 1990; Gleeson and Tilman, 1992), an increase in the proportional biomass allocated to roots would be expected in parents growing under low nutrient conditions. Furthermore, trans-generational plasticity predicts that the plastic response experienced by the parental generation could be transferred to their offspring generations, as a mechanism to facilitate offspring establishment (Latzel et al., 2014; Dong et al., 2017, 2018a). Therefore, it is reasonable to predict that parent ramets of *P. stratiotes* growing under low nutrients would have increased the root production, and this plastic response would have been transmitted to the subsequent offspring generations (F1, F2, and F3). In this situation, offspring ramets having a higher proportion of roots and growing under high nutrients would be able to make better use of them and achieve maximum nutrient acquisition efficiency, which translates into the highest growth. Unfortunately, leaf, stem, and root of *P. stratiotes* were harvested together, and biomass allocation ratios were not available in our study. Therefore, to truly test this plausible explanation, studies should consider not only parental effects in terms of biomass production but also in terms of biomass allocation ratio.

Our finding was opposite to previous studies showing that the high-quality offspring produced in favorable parental environments benefited subsequent growth of offspring (Zas et al., 2013; Dong et al., 2018a, 2019; Portela et al., 2020), as stated by the “silver-spoon” effect, where parent plant growing in favorable conditions can provide more resources to their offspring (Roach and Wulff, 1987). In addition, our results also do not fit with another potential benefit of parental effects, which states that offspring generations could gain an advantage when establishing in the same or similar conditions to those experienced by their parents (Dong et al., 2017, 2018a).

The “silver-spoon” effect was detected when the F3 offspring ramets of *P. stratiotes* were grown under low nutrients but was absent when the F1 and F2 offspring ramets were grown under low nutrients. Also, the magnitude of parental nutrient effects on the subsequent growth of the F1, F2, and F3 offspring ramets in high nutrients were similar (Figures 3–5). These results do not support the third hypothesis that the magnitude of parental effects would decrease with increasing vegetative offspring generations. This is very likely because the difference in the size advantage of offspring of different generations cannot transmit to their growth benefits, as discussed above.

## Conclusions

We conclude that nutrient-induced clonal parental effects can influence offspring growth of different vegetative generations, suggesting that clonal parental effects can transmit fast. However, heavier and, likely, bigger ramets produced under high nutrients in the parental generation could not increase the subsequent growth of offspring. Thus, parental provisioning in favorable conditions may not always increase the growth of their offspring, partly depending on root allocation but not ramet biomass.

## Data Availability Statement

The raw data supporting the conclusions of this article will be made available by the authors, without undue reservation.

## Author Contributions

L-MZ and F-HY designed the experiment. L-MZ, J-FZ, W-HY, C-YQ, and D-HW performed the experiment and collected data. L-MZ and J-FZ analyzed the data. L-MZ wrote the first draft of the manuscript. F-HY and SR contributed substantially to the revisions. All authors contributed to the article and approved the submitted version.

## Funding

The research was supported by the National Natural Science Foundation of China (Grants 32071527 and 32101261).

## Conflict of Interest

The authors declare that the research was conducted in the absence of any commercial or financial relationships that could be construed as a potential conflict of interest.

## Publisher's Note

All claims expressed in this article are solely those of the authors and do not necessarily represent those of their affiliated organizations, or those of the publisher, the editors and the reviewers. Any product that may be evaluated in this article, or claim that may be made by its manufacturer, is not guaranteed or endorsed by the publisher.
