# Supplementary figures and images for: Clonal Parental Effects on Offspring Growth of Different Vegetative Generations in the Aquatic Plant Pistia stratiotes
Source: Front Plant Sci. 2022 Jun 27;13:890309. doi: 10.3389/fpls.2022.890309 (PMC9272891; doi:10.3389/fpls.2022.890309)

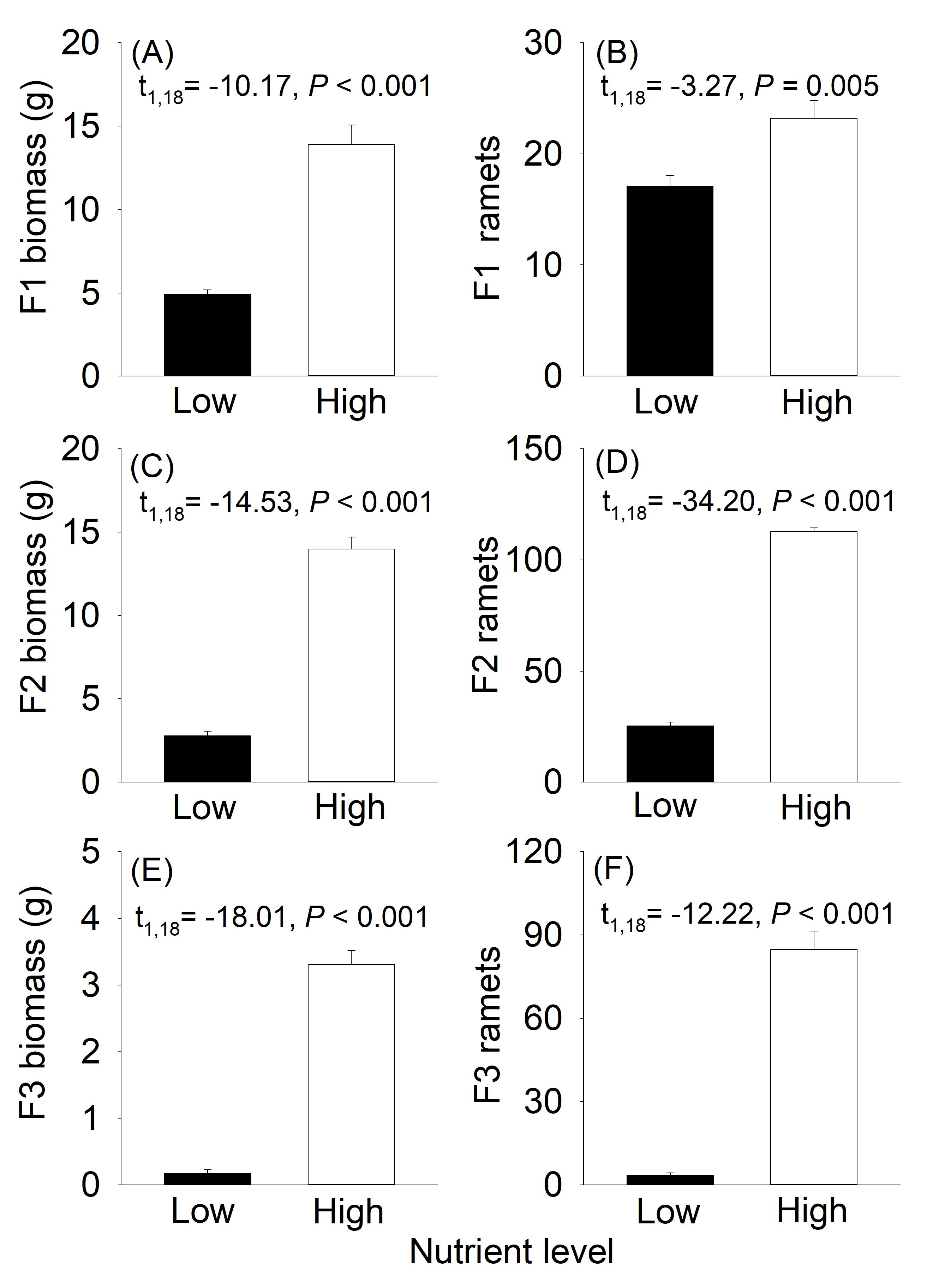

Supplement: Supplementary file 1 [file Image_1.jpg]
